# Barack Obama Blindness (BOB): Absence of Visual Awareness to a Single Object

**DOI:** 10.3389/fnhum.2016.00118

**Published:** 2016-03-22

**Authors:** Marjan Persuh, Robert D. Melara

**Affiliations:** ^1^Department of Social Sciences, Human Services and Criminal Justice, Borough of Manhattan Community College, City University of New YorkNew York, NY, USA; ^2^Department of Psychology, City College, City University of New YorkNew York, NY, USA

**Keywords:** perceptual blindness, top-down processing, awareness, visual perception, consciousness

## Abstract

In two experiments, we evaluated whether a perceiver’s prior expectations could alone obliterate his or her awareness of a salient visual stimulus. To establish expectancy, observers first made a demanding visual discrimination on each of three baseline trials. Then, on a fourth, critical trial, a single, salient and highly visible object appeared in full view at the center of the visual field and in the absence of any competing visual input. Surprisingly, fully half of the participants were unaware of the solitary object in front of their eyes. Dramatically, observers were blind even when the only stimulus on display was the face of U.S. President Barack Obama. We term this novel, counterintuitive phenomenon, Barack Obama Blindness (BOB). Employing a method that rules out putative memory effects by probing awareness immediately after presentation of the critical stimulus, we demonstrate that the BOB effect is a true failure of conscious vision.

## Introduction

Visual perception arises not only from external stimulus information impinging on photoreceptors in the retina, but also from top-down processes—including attention, expectation, and task set—that interact with incoming visual information (Gilbert and Li, 2013). Top-down processes bias competition between objects presented simultaneously in the visual field (Desimone and Duncan, 1995). Conversely, in the absence of sensory competition, as when an object appears in isolation, the cognitive processes that bias competition are dispensable, suggesting that an isolated object would invariably reach a perceiver’s awareness (van Boxtel et al., 2010). In the current study we examined this prediction, while also manipulating perceivers’ prior expectations, to explore the conditions under which conscious perception of salient visual objects is eliminated.

Numerous studies have demonstrated that attention alters the appearance of consciously perceived objects (Carrasco et al., 2004; Gobell and Carrasco, 2005; Liu et al., 2006). The most convincing of these behavioral results emerges from experiments investigating inattentional blindness (IB; Rock et al., 1992; Mack and Rock, 1998). IB is a failure to consciously perceive otherwise fully visible objects once another activity engages attention. In IB experiments, participants perform an attentionally demanding task during a set of baseline trials. Then, on the critical trial, a new object unexpectedly appears together with the stimuli comprising the primary task. A significant proportion of participants fails to notice the new object, particularly (and interestingly) when the object appears at the center of the visual field (Mack and Rock, 1998).

Although several early IB studies presented the critical object in near isolation (Rock et al., 1992), to the best of our knowledge in no studies has the critical trial been restricted to a display of the novel object alone. Thus, no previous IB study has been able to unambiguously distinguish the role of top-down influences from the effects of sensory competition alone on the appearance of visual objects. The current study fills this important gap. Subsequent IB studies (including from our laboratory) explored different subtypes of top-down influences on IB, including spatial IB (Newby and Rock, 1998) and feature-based IB (Persuh et al., 2014). Yet the fundamental limitation of the original and all subsequent IB studies is that the unexpected object appears alongside stimuli from the primary attention task. In correcting this limitation in the current study we also are able to address an alternative interpretation of IB, namely, that the failure to report novel objects signifies forgetting, that is inattentional amnesia, rather than true perceptual blindness (Wolfe, 1999).

The IB paradigm is well suited to examine contributions on conscious perception from both stimulus characteristics and top-down influences. Although certain stimulus characteristics, such as unique color or sudden onset, increase the probability that a novel object will be consciously perceived, in fact the major factor determining conscious perception is the observer’s attentional set (Most et al., 2005). Participants engaged in an attentionally demanding activity, for example, are more likely to detect an unexpected object when its features (e.g., brightness) resemble those of attended objects (Most et al., 2001), even when the shared features are semantic (Koivisto and Revonsuo, 2007; Most, 2013).

Behavioral experiments using IB align with recent results from neurophysiological research. Influences on visual perception from top-down processing have been extensively documented across all stages of the visual hierarchy (Gilbert and Sigman, 2007; Gilbert and Li, 2013). These influences can spread as far back as the lateral geniculate nucleus (O’Connor et al., 2002; McAlonan et al., 2008). Recent evidence suggests that top-down processes reach the level of individual neurons, shaping receptive fields in primary visual cortex (Li et al., 2004). Top-down expectations can even alter cell properties and modify selectivity across a population of V1 superficial layer neurons (McManus et al., 2011). Such neural influences have been observed even before the stimulus presentation (Kastner et al., 1999; Ress et al., 2000).

The neurophysiological findings suggest that, in creating a set of filters in early visual areas to calibrate tuning properties, expectation and other top-down processes aid in resolving competition between two or more objects (Desimone and Duncan, 1995). The use of competing stimuli in visual neurophysiological research is ubiquitous because, without competition, top-down modulation is largely unnecessary (Reynolds and Chelazzi, 2004). Indeed, neurophysiological evidence suggests that when presented in isolation, perceivers in any attentional state will always become aware of the isolated object (van Boxtel et al., 2010). Yet this hypothesis has never been tested directly in either behavioral or neurophysiological research. That is exactly our approach in the current behavioral study. We tested naive participants in a modified version of the classic IB paradigm (Mack and Rock, 1998). Participants first performed a series of baseline trials to establish expectations about upcoming stimuli. Then, on the critical trial, only the novel object appeared at the center of the display. We asked whether participants fail to consciously perceive a single, isolated object in the absence of any sensory competition.

## Experiment 1

We tested whether the repeated exposure in an attentionally demanding task to a specific category of objects creates an expectation in which a highly salient object fails to reach awareness, even when presented in the absence of other objects. Participants first performed three (baseline) trials of a gender comparison task to pairs of faces in the visual periphery. Then, on the critical trial, a salient (non-face) object appeared alone at the center of the display, without faces or any other stimuli on the screen. We asked whether participants consciously perceived this novel object.

### Materials and Methods

#### Participants

Twenty participants (11 females), between ages 18–24 (*M* = 20.1 years), were tested after providing informed consent. Sample size was based on estimated power from a previous pilot experiment. The Institutional Review Board of the City University of New York approved the protocol. All participants were recruited from the undergraduate subject pool of The City College of New York, receiving course credit for participation. All participants had normal or corrected-to-normal vision and were free of any neurological diseases.

#### Stimuli and Apparatus

Participants were seated in a dimly lit, sound attenuated booth. A chin rest, positioned 57 cm from a CRT monitor with a refresh rate of 100 Hz, was used to prevent head movements. Stimuli were two faces, subtending 5.5°, presented 6.4° from a central, white fixation dot, subtending 0.3°. Faces were extracted from a database of face stimuli, varying along a male-to-female continuum (Zhao et al., 2011). The novel object, presented at the center of the display on critical trials, was one of the four shapes—square, circle, diamond or star—each subtending 1° (*L* = 0 cd/m^2^) . A visual mask, subtending 18.2°, comprised black lines in random orientations. Stimuli appeared on a gray background (*L* = 13 cd/m^2^) .

#### Procedure

**Figure 1:**
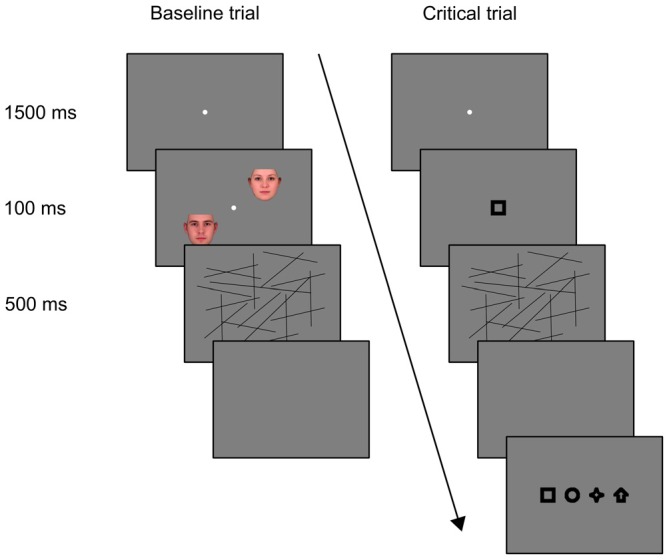
**A diagram of the trial sequence in Experiment 1.** Each participant was first presented with three baseline trials, followed by the critical trial. The last, control trial was identical to the critical trial.

Each session consisted of five experimental trials, preceded by four baseline practice trials. Participants were instructed to fixate a dot in the center of the display and to determine whether a pair of faces was same or different in gender. Each trial began with the fixation dot presented for 1500 ms. Then, on each of the three baseline trials, the two faces appeared diagonally across the screen for 100 ms, followed by a visual mask for 500 ms (Figure 1). The diagonal and the gender of each face were determined randomly on each trial. Participants reported their responses verbally, which were entered manually by an experimenter. On the fourth, critical trial, no faces were presented; instead, one of the four shapes (square, arrow, star or circle) appeared at the center of display for 100 ms, followed by the visual mask for 500 ms. Each shape was presented with equal frequency across participants. Immediately, the participant was asked whether or not anything new appeared on the screen, and his or her response was recorded. The participant was then told that indeed a new shape had appeared. The participant was shown the four possible shapes, presented horizontally side-by-side at the center of the display, and asked to select the actual shape. The shapes remained on the screen until the participant made a decision, which the experimenter recorded. Order of the four shapes on the screen was random for each participant. A final, fifth trial, the control trial, was identical to the prior critical trial; the shape presented on the critical trial was presented again. The participant was again asked if anything new appeared on the screen, and then asked to select the new object among the four possible shapes.

### Results

**Figure 2:**
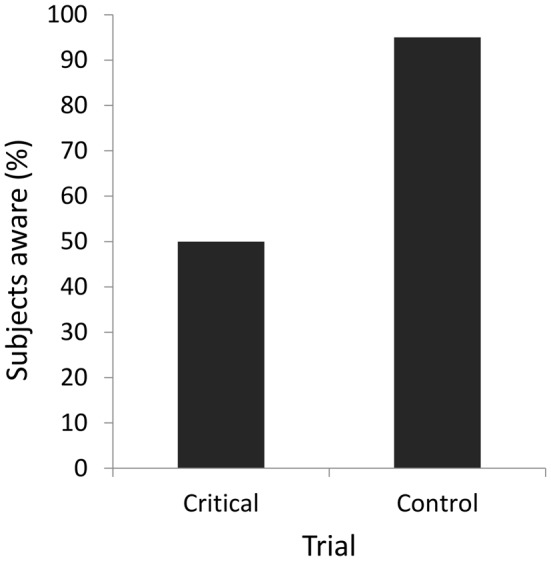
**Percentage of participants consciously perceiving the novel object in the critical and control trials in Experiment 1**.

Participants found gender discrimination in the baseline trials difficult, with performance averaging 61.7%. In answer to the question of whether they perceived anything new on the critical trial, fully half of the participants replied that they had not noticed a novel object (Figure 2). Of those participants, only two (20%), a chance-level value, correctly identified the novel object in a forced-choice task. Of the participants who acknowledged a new object, most (90%) were able to identify the object correctly in the forced-choice procedure. On the fifth, control trial, almost all of the participants (95%) now admitted being aware of a new object, a percentage significantly outpacing awareness in the critical trial (McNemar’s exact test, *p* = 0.004); most of the participants who were aware of the new object also were able to select it in the forced-choice procedure (89.5%).

## Experiment 2

Experiment 1, which employed a demanding discrimination task in baseline trials, revealed a significant degree of blindness to geometric shapes presented at the center of the display in the absence of any competing visual input. Yet it is possible that blindness to isolated objects only manifests with such strong top-down influences. In Experiment 2, we employed an easier baseline discrimination task, and we presented on the critical trial a novel object that was truly engaging: the face of President Barack Obama. We asked whether blindness persists even for such a highly familiar stimulus in the context of a less demanding discrimination task.

### Materials and Methods

#### Participants

Twenty students (10 females), between ages 18–35 (*M* = 20.7 years), recruited from the undergraduate subject pool of The City College of New York, were tested after consenting to participate and received course credit for participation. Sample size was based on estimated power from a previous pilot experiment. All participants had normal or corrected-to-normal vision and were free of any neurological diseases.

#### Stimuli, Apparatus and Procedure

**Figure 3:**
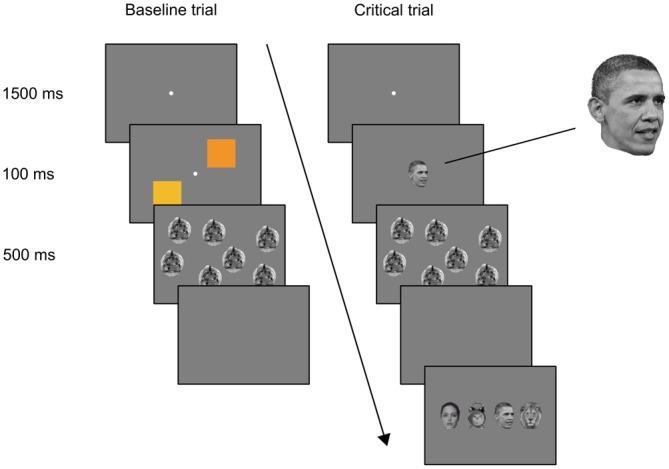
**A diagram of the trial sequence in Experiment 2.** Each participant was presented first with three baseline trials, followed by the critical trial. The last, control trial was identical to the critical trial. The order of forced-choice alternatives was randomized; in this example, from left to right: Angelina Jolie, alarm clock, Barack Obama, lion head.

Stimuli, apparatus and procedures were similar to Experiment 1, except for the following modifications. On baseline trials, two orange squares (*x* = 0.49, *y* = 0.44, *L* = 29 cd/m^2^ and *x* = 0.45, *y* = 0.47, *L* = 39 cd/m^2^), subtending 4.7°, were presented in the periphery of the display (Figure 3). On the critical (fourth) and control (fifth) trials, the face of President Barack Obama, subtending 1°, was presented at the center of the display. The forced-choice display contained the face of Barack Obama, the face of Angelina Jolie, a head of a lion, and the face of a clock. Each subtended approximately 1° of visual angle. The visual mask comprised multiple circular stimuli, obtained by superimposing rotated and transparent versions of different objects. The trial structure was identical to Experiment 1. Participants performed same/different color discrimination on three baseline trials. On the critical trial and control trials, the face of President Barack Obama was presented to every participant, followed by the forced-choice identification.

### Results

**Figure 4:**
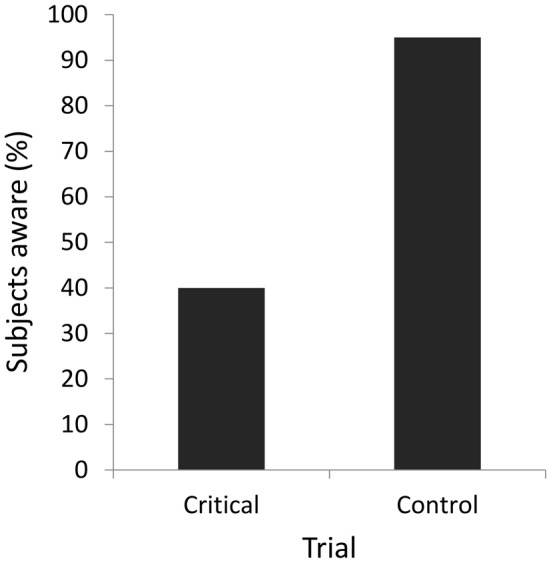
**Percentage of participants consciously perceiving the novel object in the critical and control trials in Experiment 2**.

Performance on color discrimination during the baseline trials was high, averaging 85%. When asked on the critical trial whether they noticed anything new, astonishingly, fully 60% of participants failed to notice the engaging face of Barack Obama directly before their eyes (Figure 4). Indeed, only 8.3% of participants who responded “no” correctly selected Barack Obama’s face in forced choice, a value lower than chance level. Only 40% of participants acknowledged a new object; among participants who perceived a face (4), all were able to select the President’s face in a forced-choice procedure. Four participants reported seeing something and all selected the clock in the forced-choice procedure. By contrast, almost all of the participants responded “yes” on the control trial (95%), significantly exceeding awareness on the critical trial (McNemar’s exact test, *p* = 0.001); almost all of these (94.7%) correctly selected the President’s face among the alternatives.

## Discussion

In two experiments, we demonstrated an extreme example of blindness to a single, centrally presented stimulus in the absence of sensory competition. Participants in both experiments established expectations about stimuli in the upcoming trial and so failed to consciously perceive a novel, salient stimulus when these expectations were violated. Experiment 2 provided especially dramatic evidence of blindness because here a highly meaningful and familiar stimulus, the face of President Barack Obama, appeared alone on the screen in the absence of any competing stimuli and in the context of a relatively easy color discrimination task. The Barack Obama Blindness (BOB) effect sheds new light on the role of top-down processes in visual perception, providing evidence for unanticipated limitations of conscious vision.

Many previous studies have, like ours, examined the role of attentional set in IB (Most et al., 2001; Koivisto and Revonsuo, 2007; Most, 2013). One recent study examined the role of task relevance using a simple two-object display composed of colored circle surrounded by a differently colored ring (Eitam et al., 2013). Although observers expected the stimuli, and were able to report task-relevant color, up to 25% were unable to report the color of the task-irrelevant stimulus. Authors suggested that observers likely perceived both colors but were perhaps unable to conceptualize the irrelevant color thus displaying intentional amnesia.

Ours is the first study to present a single, unexpected stimulus in isolation on the critical trial. This is significant because it has been suggested that top-down mechanisms are required to resolve competition between items in the visual field, and become progressively less critical when only few objects are present. Consequently, it has been assumed that for conscious experience of a single, isolated object there is no need to engage such mechanisms (van Boxtel et al., 2010). The BOB effect strongly suggests otherwise: when an observer’s expectations are violated, even a single object will not be perceived, despite robust sensory evidence. Even IB experiments in which the critical object appears in *near* isolation are unable to unambiguously rule out the effects of sensory competition over violations of expectation, as shown here. In other attention paradigms, such as attentional blink, blindness occurs to isolated targets presented briefly, as here, but only after participants are first shown another target stimulus (~200 ms prior) and then are fully expecting the critical second target stimulus to appear (Raymond et al., 1992).

One common criticism of IB studies is that the failure to report an unexpected stimulus may reflect a failure of memory rather than a failure of awareness. On this account, IB is actually inattentional amnesia (Wolfe, 1999). Yet the criticism only applies if participants must first make a response to the primary stimuli, during which time memory of the unexpected stimuli may fade. In our modification of the traditional IB paradigm (Mack and Rock, 1998; Cartwright-Finch and Lavie, 2007) participants never made responses on the critical trial, instead being asked immediately about the novel stimulus. Thus, the failure of our participants to report the new stimulus demonstrates a true absence of conscious perception, and not mere forgetting.

Although the percentage of unaware participants in Experiment 1 who selected the correct shape during the forced-choice procedure was at a level expected by chance, the percentage of unaware participants in Experiment 2 who correctly selected Barack Obama during forced choice was lower than chance. A probable explanation for this observation is participants’ response bias: presented with four possible objects, participants likely assumed that they would be the least likely to miss the face of the American president and so opted for one of the other three possibilities.

In conclusion, our study is the first to provide evidence of a profound effect of top-down processes on unexpected stimuli in the absence of sensory competition, here termed the BOB effect. Our study shows the fundamental role of top-down processes in establishing perceptual awareness for single objects. These processes are instrumental for perception of even highly familiar faces, such as that of President Barack Obama.

## Author Contributions

MP developed the study concept and collected and analyzed data. MP and RDM contributed to study design and data interpretation. MP and RDM wrote the manuscript and approved the final version of the manuscript for submission.

## Conflict of Interest Statement

The authors declare that the research was conducted in the absence of any commercial or financial relationships that could be construed as a potential conflict of interest.
